# Supplementary material for: A Likelihood Approach for Real-Time Calibration of Stochastic Compartmental Epidemic Models
Source: PLoS Comput Biol. 2017 Jan 17;13(1):e1005257. doi: 10.1371/journal.pcbi.1005257 (PMC5240920; doi:10.1371/journal.pcbi.1005257)
Supplement: S1 File — (TAR.GZ) [file pcbi.1005257.s014.tar.gz › HSPH_Online-SI-Revision/PF08/n1-extreme/n1-extreme_PF2_table4.pdf]

1	newly 3 weeks spec	16.	31.79	{13., 58.}	106.438	0.286126	newly 3 weeks cumul	86.	119.48	{65., 216.}	51.7209	0.170263	newly 5 weeks spec	10.	19.15	{7., 37.}	108.7	0.322736	newly 5 weeks cumul	112.	163.52	{85., 301.}	58.0714	0.186564	newly 56 weeks cumul	131.	243.05	{109., 441.}	94.5115	0.257107
2	newly 3 weeks spec	15.	21.93	{7., 38.}	72.7333	0.250101	newly 3 weeks cumul	66.	80.64	{26., 134.}	52.5758	0.209716	newly 5 weeks spec	14.	15.53	{4., 29.}	55.6429	0.266132	newly 5 weeks cumul	87.	114.38	{38., 192.}	58.6667	0.218775	newly 52 weeks cumul	117.	179.39	{56., 295.}	77.0171	0.257251
3	newly 3 weeks spec	43.	28.7	{10., 52.}	43.8605	0.287889	newly 3 weeks cumul	138.	104.47	{44., 183.}	40.1522	0.240516	newly 5 weeks spec	26.	19.91	{6., 39.}	47.9615	0.306477	newly 5 weeks cumul	184.	147.99	{63., 263.}	40.3641	0.231524	newly 54 weeks cumul	247.	243.12	{92., 419.}	43.9676	0.218743
4	newly 3 weeks spec	35.	22.2	{5., 50.}	51.4286	0.388562	newly 3 weeks cumul	105.	79.84	{27., 149.}	44.2095	0.266531	newly 5 weeks spec	26.	14.74	{2., 31.}	52.4615	0.491485	newly 5 weeks cumul	155.	112.84	{33., 211.}	45.1097	0.287634	newly 53 weeks cumul	252.	188.53	{46., 372.}	47.6389	0.315732
5	newly 3 weeks spec	18.	11.33	{2., 23.}	53.0556	0.455264	newly 3 weeks cumul	78.	41.03	{8., 76.}	51.4744	0.428147	newly 5 weeks spec	14.	7.98	{1., 15.}	56.8571	0.490706	newly 5 weeks cumul	116.	59.04	{13., 110.}	53.1897	0.444112	newly 46 weeks cumul	161.	102.53	{15., 211.}	53.3975	0.432983
6	newly 3 weeks spec	29.	19.35	{4., 40.}	50.1724	0.365698	newly 3 weeks cumul	76.	61.92	{21., 116.}	47.1053	0.268889	newly 5 weeks spec	16.	14.1	{2., 31.}	60.25	0.395488	newly 5 weeks cumul	103.	93.38	{28., 184.}	48.9515	0.259996	newly 51 weeks cumul	158.	189.36	{42., 406.}	70.1772	0.297639
7	newly 3 weeks spec	23.	15.22	{2., 29.}	52.9565	0.447363	newly 3 weeks cumul	107.	56.96	{14., 92.}	53.514	0.420602	newly 5 weeks spec	18.	11.	{1., 20.}	54.5556	0.510454	newly 5 weeks cumul	144.	80.39	{16., 133.}	51.3125	0.408022	newly 55 weeks cumul	190.	129.21	{19., 234.}	49.4263	0.386879
8	newly 3 weeks spec	21.	14.01	{1., 29.}	51.1905	0.423568	newly 3 weeks cumul	65.	51.7	{10., 91.}	42.4615	0.301176	newly 5 weeks spec	12.	10.3	{1., 22.}	56.3333	0.447092	newly 5 weeks cumul	85.	74.18	{11., 132.}	43.2471	0.295449	newly 49 weeks cumul	108.	123.21	{12., 259.}	64.0833	0.345127
9	newly 3 weeks spec	14.	32.31	{16., 52.}	135.357	0.330397	newly 3 weeks cumul	73.	113.75	{59., 193.}	64.7534	0.197117	newly 5 weeks spec	7.	21.73	{8., 38.}	215.286	0.441494	newly 5 weeks cumul	94.	162.73	{91., 263.}	79.2234	0.22562	newly 56 weeks cumul	112.	255.94	{121., 396.}	133.857	0.330485
10	newly 3 weeks spec	36.	13.42	{1., 29.}	63.3333	0.639407	newly 3 weeks cumul	109.	47.8	{10., 87.}	57.5229	0.498222	newly 5 weeks spec	14.	9.48	{1., 20.}	57.7143	0.513184	newly 5 weeks cumul	147.	68.42	{12., 132.}	55.5782	0.48413	newly 49 weeks cumul	210.	115.26	{12., 251.}	57.1238	0.498067
11	newly 3 weeks spec	14.	22.63	{6., 41.}	88.5	0.264901	newly 3 weeks cumul	74.	77.74	{33., 138.}	48.9189	0.208943	newly 5 weeks spec	6.	15.58	{2., 30.}	184.333	0.437375	newly 5 weeks cumul	95.	111.96	{41., 196.}	56.8211	0.221029	newly 55 weeks cumul	108.	185.15	{53., 426.}	98.8241	0.279815
12	newly 3 weeks spec	26.	36.83	{16., 62.}	61.7308	0.228594	newly 3 weeks cumul	91.	124.48	{65., 221.}	52.7473	0.179037	newly 5 weeks spec	9.	24.66	{6., 50.}	187.778	0.435	newly 5 weeks cumul	114.	179.36	{89., 319.}	69.4035	0.214905	newly 56 weeks cumul	127.	290.25	{127., 544.}	136.37	0.33763
13	newly 3 weeks spec	35.	12.16	{2., 22.}	65.6	0.642818	newly 3 weeks cumul	94.	41.73	{10., 72.}	56.7766	0.484059	newly 5 weeks spec	33.	8.81	{1., 18.}	73.7879	0.830971	newly 5 weeks cumul	163.	61.13	{15., 107.}	63.0982	0.570674	newly 49 weeks cumul	326.	108.44	{20., 220.}	67.	0.668072
14	newly 3 weeks spec	7.	21.46	{7., 37.}	214.571	0.468712	newly 3 weeks cumul	41.	77.1	{29., 130.}	100.732	0.283401	newly 5 weeks spec	2.	14.07	{3., 26.}	608.5	0.762618	newly 5 weeks cumul	46.	108.08	{41., 170.}	143.304	0.351317	newly 53 weeks cumul	53.	170.73	{45., 318.}	230.698	0.455176
15	newly 3 weeks spec	12.	11.88	{1., 23.}	60.1667	0.430199	newly 3 weeks cumul	65.	42.54	{7., 77.}	47.3846	0.418173	newly 5 weeks spec	12.	8.82	{0., 19.}	57.5	0.514705	newly 5 weeks cumul	89.	62.	{9., 119.}	47.9101	0.425988	newly 48 weeks cumul	106.	109.74	{10., 237.}	67.8302	0.458307
16	newly 3 weeks spec	31.	18.3	{1., 37.}	54.4516	0.520395	newly 3 weeks cumul	81.	66.27	{7., 135.}	49.642	0.37295	newly 5 weeks spec	20.	12.98	{0., 30.}	59.3	0.636128	newly 5 weeks cumul	118.	94.37	{9., 194.}	51.4322	0.404788	newly 55 weeks cumul	180.	155.22	{12., 327.}	56.2889	0.439031
17	newly 3 weeks spec	33.	19.53	{6., 34.}	48.0303	0.382284	newly 3 weeks cumul	111.	70.37	{28., 125.}	43.955	0.295781	newly 5 weeks spec	26.	14.76	{3., 31.}	52.	0.453693	newly 5 weeks cumul	160.	102.88	{41., 176.}	43.4	0.297753	newly 52 weeks cumul	266.	193.29	{52., 364.}	49.6805	0.34423
18	newly 3 weeks spec	26.	30.44	{12., 51.}	52.	0.217115	newly 3 weeks cumul	118.	108.65	{49., 177.}	39.1441	0.196677	newly 5 weeks spec	13.	21.08	{7., 39.}	85.8462	0.287091	newly 5 weeks cumul	153.	154.82	{69., 257.}	40.549	0.189354	newly 55 weeks cumul	200.	242.96	{93., 430.}	52.38	0.211374
19	newly 3 weeks spec	32.	20.87	{3., 41.}	54.4688	0.462147	newly 3 weeks cumul	144.	75.98	{15., 148.}	55.2222	0.451117	newly 5 weeks spec	11.	14.23	{0., 27.}	82.8182	0.482267	newly 5 weeks cumul	177.	106.87	{21., 200.}	52.9435	0.423093	newly 56 weeks cumul	231.	167.	{23., 312.}	51.3593	0.409115
20	newly 3 weeks spec	8.	11.68	{1., 24.}	89.75	0.434209	newly 3 weeks cumul	57.	44.51	{7., 86.}	47.	0.308648	newly 5 weeks spec	5.	7.5	{0., 17.}	101.6	0.495333	newly 5 weeks cumul	69.	61.89	{9., 109.}	50.5072	0.30816	newly 49 weeks cumul	94.	97.93	{10., 213.}	64.3936	0.351303
21	newly 3 weeks spec	40.	19.48	{5., 35.}	55.75	0.449016	newly 3 weeks cumul	123.	68.3	{20., 131.}	50.4878	0.371505	newly 5 weeks spec	31.	14.05	{1., 28.}	59.5806	0.586677	newly 5 weeks cumul	179.	98.82	{22., 184.}	50.905	0.385669	newly 52 weeks cumul	267.	183.14	{30., 345.}	49.7828	0.372049
22	newly 3 weeks spec	22.	22.49	{8., 41.}	54.3182	0.264553	newly 3 weeks cumul	81.	77.86	{33., 152.}	45.358	0.217957	newly 5 weeks spec	11.	16.13	{3., 33.}	87.5455	0.351508	newly 5 weeks cumul	100.	112.55	{43., 214.}	52.69	0.220629	newly 53 weeks cumul	148.	195.65	{51., 406.}	75.7095	0.290567
23	newly 3 weeks spec	17.	19.12	{3., 42.}	63.6471	0.337378	newly 3 weeks cumul	88.	72.42	{15., 151.}	46.2727	0.305027	newly 5 weeks spec	9.	13.35	{2., 29.}	90.5556	0.388171	newly 5 weeks cumul	105.	101.21	{22., 211.}	51.5714	0.296907	newly 55 weeks cumul	155.	157.51	{31., 298.}	54.5613	0.308815
24	newly 3 weeks spec	14.	28.01	{10., 47.}	110.357	0.285977	newly 3 weeks cumul	65.	103.06	{53., 159.}	66.6154	0.203313	newly 5 weeks spec	7.	18.2	{7., 35.}	165.714	0.356819	newly 5 weeks cumul	79.	144.4	{69., 227.}	88.3544	0.245508	newly 56 weeks cumul	97.	217.66	{88., 392.}	129.278	0.305252
25	newly 3 weeks spec	37.	24.99	{12., 46.}	43.3784	0.294664	newly 3 weeks cumul	118.	85.78	{39., 142.}	39.6949	0.238436	newly 5 weeks spec	14.	18.1	{4., 33.}	64.1429	0.282071	newly 5 weeks cumul	153.	125.48	{56., 208.}	38.0654	0.216422	newly 53 weeks cumul	253.	228.42	{76., 396.}	41.7628	0.231013
26	newly 3 weeks spec	7.	26.15	{10., 46.}	276.143	0.526585	newly 3 weeks cumul	46.	102.24	{53., 148.}	124.783	0.322175	newly 5 weeks spec	5.	16.93	{4., 33.}	252.2	0.532118	newly 5 weeks cumul	56.	140.49	{69., 211.}	153.411	0.367748	newly 55 weeks cumul	70.	192.04	{81., 335.}	177.2	0.389348
27	newly 3 weeks spec	41.	14.84	{2., 29.}	64.878	0.638465	newly 3 weeks cumul	120.	53.36	{14., 102.}	57.4167	0.487661	newly 5 weeks spec	16.	10.57	{1., 22.}	58.0625	0.519659	newly 5 weeks cumul	166.	77.01	{19., 144.}	57.0663	0.491511	newly 50 weeks cumul	260.	139.34	{20., 301.}	57.9615	0.506701
28	newly 3 weeks spec	13.	21.	{7., 34.}	81.6923	0.265015	newly 3 weeks cumul	77.	76.71	{33., 115.}	38.5325	0.184449	newly 5 weeks spec	17.	15.2	{2., 29.}	52.	0.347606	newly 5 weeks cumul	109.	110.14	{45., 172.}	41.4679	0.201388	newly 54 weeks cumul	162.	183.02	{63., 372.}	58.716	0.261674
29	newly 3 weeks spec	7.	19.17	{7., 31.}	180.714	0.417168	newly 3 weeks cumul	71.	70.69	{31., 105.}	35.8732	0.179375	newly 5 weeks spec	8.	11.94	{3., 21.}	82.5	0.323486	newly 5 weeks cumul	91.	97.96	{40., 149.}	40.3956	0.189204	newly 51 weeks cumul	100.	147.84	{54., 270.}	69.26	0.241163
30	newly 3 weeks spec	10.	7.38	{0., 17.}	57.6	0.555772	newly 3 weeks cumul	42.	27.37	{3., 56.}	51.3571	0.434422	newly 5 weeks spec	6.	5.46	{0., 13.}	72.	0.589985	newly 5 weeks cumul	53.	39.2	{3., 78.}	52.6038	0.433985	newly 46 weeks cumul	78.	71.32	{3., 151.}	68.7179	0.504139
31	newly 3 weeks spec	10.	28.09	{9., 45.}	187.1	0.411636	newly 3 weeks cumul	87.	100.8	{48., 155.}	41.7011	0.167184	newly 5 weeks spec	4.	19.18	{6., 36.}	384.	0.621839	newly 5 weeks cumul	99.	142.53	{66., 216.}	60.596	0.201957	newly 55 weeks cumul	122.	222.37	{85., 395.}	96.1885	0.270759
32	newly 3 weeks spec	6.	29.44	{10., 51.}	394.333	0.628839	newly 3 weeks cumul	62.	102.9	{48., 167.}	76.5484	0.228358	newly 5 weeks spec	6.	20.03	{3., 39.}	247.833	0.485498	newly 5 weeks cumul	78.	147.29	{66., 238.}	97.4231	0.268304	newly 55 weeks cumul	82.	235.39	{87., 461.}	193.963	0.413003
33	newly 3 weeks spec	35.	20.42	{5., 40.}	49.0286	0.393409	newly 3 weeks cumul	123.	72.51	{25., 138.}	47.439	0.345822	newly 5 weeks spec	29.	14.87	{3., 31.}	54.8621	0.482709	newly 5 weeks cumul	183.	104.55	{39., 193.}	48.224	0.361341	newly 51 weeks cumul	278.	182.09	{45., 352.}	46.3849	0.350702
34	newly 3 weeks spec	27.	26.33	{7., 48.}	48.5556	0.263014	newly 3 weeks cumul	98.	92.5	{36., 163.}	42.3878	0.211534	newly 5 weeks spec	12.	19.21	{3., 35.}	98.0833	0.405929	newly 5 weeks cumul	137.	133.73	{48., 225.}	44.0219	0.218321	newly 54 weeks cumul	165.	223.15	{61., 405.}	70.5879	0.268055
35	newly 3 weeks spec	25.	28.91	{11., 53.}	50.6	0.211813	newly 3 weeks cumul	103.	95.16	{41., 166.}	39.9612	0.197199	newly 5 weeks spec	20.	20.34	{5., 40.}	56.3	0.327073	newly 5 weeks cumul	144.	139.32	{58., 256.}	42.25	0.201673	newly 55 weeks cumul	192.	243.54	{81., 437.}	64.6771	0.251859
36	newly 3 weeks spec	28.	22.27	{6., 47.}	53.8214	0.351827	newly 3 weeks cumul	103.	79.92	{24., 169.}	48.5049	0.290752	newly 5 weeks spec	9.	15.92	{2., 35.}	110.889	0.36718	newly 5 weeks cumul	131.	113.9	{33., 233.}	50.2595	0.278685	newly 52 weeks cumul	175.	182.24	{47., 383.}	60.7543	0.29418
37	newly 3 weeks spec	25.	9.89	{1., 24.}	61.64	0.646686	newly 3 weeks cumul	95.	36.75	{6., 80.}	61.5895	0.610114	newly 5 weeks spec	12.	6.87	{0., 18.}	65.25	0.664951	newly 5 weeks cumul	128.	52.22	{8., 113.}	60.7813	0.617185	newly 47 weeks cumul	171.	82.82	{9., 195.}	58.8538	0.613448
38	newly 3 weeks spec	18.	23.19	{9., 41.}	60.9444	0.226423	newly 3 weeks cumul	76.	81.03	{35., 141.}	44.3553	0.191996	newly 5 weeks spec	9.	16.19	{4., 32.}	105.	0.333119	newly 5 weeks cumul	96.	115.97	{49., 207.}	52.4479	0.202996	newly 52 weeks cumul	127.	195.16	{63., 381.}	80.252	0.256894
39	newly 3 weeks spec	23.	29.52	{10., 52.}	57.3913	0.24156	newly 3 weeks cumul	103.	103.6	{54., 180.}	37.7087	0.178245	newly 5 weeks spec	6.	21.41	{5., 43.}	265.5	0.508179	newly 5 weeks cumul	136.	150.84	{70., 261.}	42.9559	0.187195	newly 55 weeks cumul	164.	253.35	{82., 498.}	79.5793	0.260884
40	newly 3 weeks spec	14.	12.32	{1., 27.}	60.4286	0.451526	newly 3 weeks cumul	66.	47.83	{6., 109.}	55.4394	0.454716	newly 5 weeks spec	15.	8.56	{0., 23.}	67.8667	0.715696	newly 5 weeks cumul	96.	66.93	{6., 164.}	57.1563	0.488135	newly 53 weeks cumul	143.	107.96	{6., 254.}	63.5944	0.530374
41	newly 3 weeks spec	19.	13.31	{2., 26.}	54.0526	0.466888	newly 3 weeks cumul	71.	44.96	{9., 82.}	48.0282	0.394834	newly 5 weeks spec	8.	9.31	{1., 20.}	77.125	0.447823	newly 5 weeks cumul	101.	64.82	{10., 122.}	48.9703	0.409418	newly 48 weeks cumul	154.	125.19	{10., 278.}	58.9026	0.449503
42	newly 3 weeks spec	21.	29.25	{12., 50.}	62.0476	0.226932	newly 3 weeks cumul	118.	109.2	{48., 169.}	36.2542	0.185861	newly 5 weeks spec	14.	19.2	{4., 36.}	73.1429	0.302359	newly 5 weeks cumul	148.	152.29	{62., 243.}	40.1014	0.188964	newly 56 weeks cumul	159.	228.06	{83., 437.}	66.8553	0.233278
43	newly 3 weeks spec	41.	30.14	{12., 55.}	45.8049	0.276488	newly 3 weeks cumul	137.	101.29	{43., 189.}	43.292	0.25557	newly 5 weeks spec	20.	21.59	{5., 39.}	56.35	0.275003	newly 5 weeks cumul	181.	148.55	{61., 259.}	42.9779	0.237599	newly 55 weeks cumul	229.	246.29	{88., 465.}	51.8384	0.23708
44	newly 3 weeks spec	21.	23.32	{8., 43.}	50.8571	0.236094	newly 3 weeks cumul	78.	84.65	{35., 145.}	45.6026	0.198077	newly 5 weeks spec	11.	17.35	{3., 33.}	93.9091	0.342863	newly 5 weeks cumul	103.	121.79	{49., 213.}	51.5243	0.20936	newly 53 weeks cumul	147.	201.26	{62., 388.}	70.4082	0.25004
45	newly 3 weeks spec	42.	29.46	{13., 50.}	42.7619	0.270312	newly 3 weeks cumul	127.	103.75	{40., 163.}	35.9134	0.201574	newly 5 weeks spec	16.	19.47	{6., 35.}	59.9375	0.271327	newly 5 weeks cumul	166.	146.94	{57., 230.}	36.241	0.195001	newly 56 weeks cumul	228.	230.78	{99., 404.}	46.5965	0.225603
46	newly 3 weeks spec	28.	24.62	{7., 42.}	45.9286	0.259912	newly 3 weeks cumul	111.	90.54	{34., 150.}	41.2252	0.234138	newly 5 weeks spec	15.	16.93	{3., 32.}	64.7333	0.342438	newly 5 weeks cumul	142.	127.19	{42., 219.}	43.0634	0.231147	newly 53 weeks cumul	196.	205.82	{56., 409.}	53.7449	0.25842
47	newly 3 weeks spec	37.	25.21	{10., 48.}	47.2703	0.304687	newly 3 weeks cumul	141.	87.09	{38., 169.}	47.7943	0.310267	newly 5 weeks spec	23.	20.01	{4., 38.}	51.087	0.327558	newly 5 weeks cumul	188.	129.56	{54., 245.}	45.3936	0.280625	newly 54 weeks cumul	283.	245.8	{83., 484.}	49.1661	0.276486
48	newly 3 weeks spec	34.	16.16	{1., 37.}	60.8235	0.64338	newly 3 weeks cumul	113.	58.89	{11., 128.}	58.7876	0.542787	newly 5 weeks spec	18.	11.46	{0., 28.}	62.4444	0.659321	newly 5 weeks cumul	163.	83.33	{13., 197.}	59.4294	0.569745	newly 54 weeks cumul	230.	131.03	{13., 330.}	61.5696	0.590614
49	newly 3 weeks spec	30.	23.73	{10., 41.}	40.3667	0.229448	newly 3 weeks cumul	104.	88.84	{42., 133.}	32.2308	0.171289	newly 5 weeks spec	16.	15.03	{4., 26.}	49.1875	0.261291	newly 5 weeks cumul	140.	122.94	{58., 190.}	33.2429	0.173594	newly 56 weeks cumul	177.	183.69	{66., 314.}	46.6271	0.207916
50	newly 3 weeks spec	29.	14.16	{2., 34.}	63.3793	0.62646	newly 3 weeks cumul	85.	50.43	{6., 107.}	55.4941	0.486832	newly 5 weeks spec	17.	9.63	{0., 20.}	64.5294	0.657225	newly 5 weeks cumul	130.	71.04	{7., 143.}	58.6154	0.540595	newly 54 weeks cumul	176.	122.18	{7., 265.}	65.0455	0.552326
